# Pneumatic Soft Actuators With Kirigami Skins

**DOI:** 10.3389/frobt.2021.749051

**Published:** 2021-09-13

**Authors:** Hesameddin Khosravi, Steven M. Iannucci, Suyi Li

**Affiliations:** Dynamic Matter Laboratory, Department of Mechanical Engineering, Clemson University, Clemson, SC, United States

**Keywords:** kirigami, pneumatic actuator, soft robotics, actuator kinematic analyses, continuum actuator

## Abstract

Soft pneumatic actuators have become indispensable for many robotic applications due to their reliability, safety, and design flexibility. However, the currently available actuator designs can be challenging to fabricate, requiring labor-intensive and time-consuming processes like reinforcing fiber wrapping and elastomer curing. To address this issue, we propose to use simple-to-fabricate kirigami skins—plastic sleeves with carefully arranged slit cuts—to construct pneumatic actuators with pre-programmable motion capabilities. Such kirigami skin, wrapped outside a cylindrical balloon, can transform the volumetric expansion from pneumatic pressure into anisotropic stretching and shearing, creating a combination of axial extension and twisting in the actuator. Moreover, the kirigami skin exhibits out-of-plane buckling near the slit cut, which enables high stretchability. To capture such complex deformations, we formulate and experimentally validates a new kinematics model to uncover the linkage between the kirigami cutting pattern design and the actuator’s motion characteristics. This model uses a virtual fold and rigid-facet assumption to simplify the motion analysis without sacrificing accuracy. Moreover, we tested the pressure-stroke performance and elastoplastic behaviors of the kirigami-skinned actuator to establish an operation protocol for repeatable performance. Analytical and experimental parametric analysis shows that one can effectively pre-program the actuator’s motion performance, with considerable freedom, simply by adjusting the angle and length of the slit cuts. The results of this study can establish the design and analysis framework for a new family of kirigami-skinned pneumatic actuators for many robotic applications.

## 1 Introduction

New inspirations from nature, advanced fabrication technologies, and the ongoing evolution in control theory have fostered the rapid development of soft robotics over the past decades (Metin, 2018; Paik, 2018; Rus and Tolley, 2018; Schmitt et al., 2018; Wallin et al., 2018; Walker et al., 2020; Terryn et al., 2021). Constructing an entirely soft robot is a multi-faceted challenge, requiring compliant and continuous robotic skeletons, sensors, power supply, and many other components. Nonetheless, a robust and simple-to-fabricate soft actuator is always a critical component. Such actuator should be lightweight, safe to operate in proximity with humans (Marchese et al., 2014; Abidi and Cianchetti, 2017; Kim et al., 2021), provide high-authority actuation (Kim et al., 2020), and preferably show multi-directional motions with mechanically programmable performance (Schaffner et al., 2018; Decroly et al., 2020). To this end, we have seen a wide variety of soft robotic actuators that operate with electrical (Shintake et al., 2018), thermal (Schönfeld et al., 2021; Terryn et al., 2021), optical (Galloway et al., 2019), and fluidic pressure stimuli (Polygerinos et al., 2017). Among them, soft pneumatic actuators remain the most widely used due to their simplicity, reliability, safety, and robustness against adverse working conditions (Walker et al., 2020). These advantages make them particularly appealing for bio-medical and rehabilitation applications (Cianchetti et al., 2018; Park et al., 2020).

A typical pneumatic actuator consists of interconnected inflatable or vacuum-actuated chambers made of soft elastomeric materials. To direct the pneumatic actuation into desired motion trajectory—such as extension/contraction, twisting, bending, and coiling—one can purposefully design the chamber geometry, adjust the chamber wall thickness (Zhou and Li, 2020), or embed constraining layers like helical fibers (Connolly et al., 2017; Xiong et al., 2021). For example, a cylindrical actuator wrapped with double-helical fibers can provide extension or contraction depending on the fiber angle, a single helical fiber can provide twisting, and tilted helical fiber can provide coiling motion (Connolly et al., 2017; Ryan et al., 2020). While promising, the fabrication of these pneumatic actuators, especially the fiber-reinforced ones, can be a lengthy and expensive process requiring expensive equipment (e.g., filament winding or braiding machine) or labor (e.g., manual fiber wrapping and then curing using 3D printed molds) (Jan and Althoefer, 2019). Such fabrication complexity also leads to inevitable uncertainty in the actuators’ performance.

**FIGURE 1:**
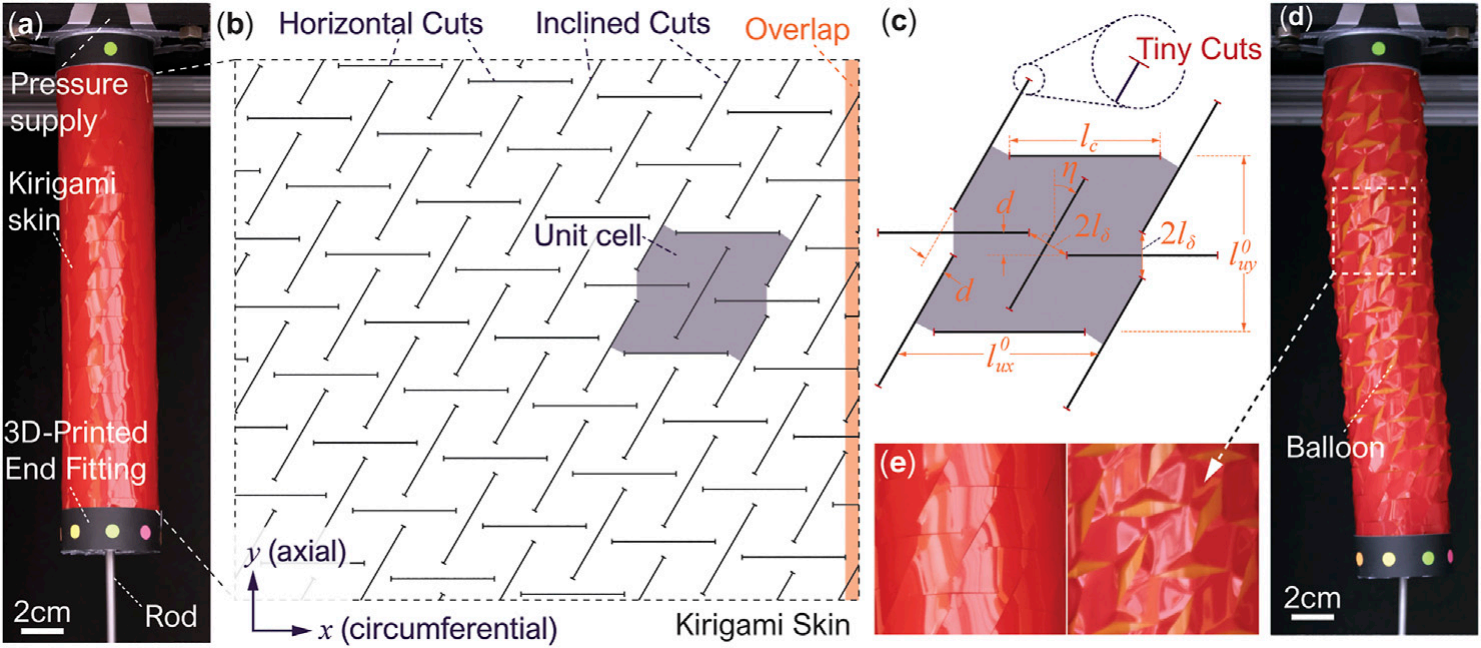
The overall concept, design, and fabrication of the kirigami-skinned actuator. **(A)** The actuator at rest, showing its different components. **(B)** The unwrapped kirigami skin showing the cut pattern. Here, a unit cell and the overlapping region for skin wrapping are highlighted. **(C)** The detailed geometry of a unit cell showing the different design parameters. Notice the additional “tiny cuts” at the tips of horizontal and inclined cuts to increase the skin’s mechanical strength. **(D)** The pressurized actuator showing both extension and twisting motion (notice the color markers’ movement on the end fitting). The slight bending is due to the additional skin stiffness from the overlapping region, and it is not considered in our kinematics model. **(E)** A close-up view showing the skin’s complex in-plane and out-of-plane deformations.

To address these issues, we propose using “kirigami skins” as a versatile and simple-to-fabricate approach to program the actuation performance of a pneumatic actuator (Figure 1). Kirigami is an ancient art of paper cutting, and it offers a unique pathway for creating 3D shapes and programmable mechanical properties from thin sheets. That is, with the addition of carefully designed cuts, a sheet material can be developed into a sophisticated 3D shape by stretching, folding, or other manipulation techniques (Abdullah et al., 2020). Meanwhile, kirigami-cut sheets can exhibit nonlinear properties such as super-stretchability (Blees et al., 2015; Tang and Yin, 2017), negative Poisson’s ratio (Hou et al., 2014; Del Broccolo et al., 2017), and stretch-induced buckling (Isobe and Okumura, 2016; Rafsanjani and Bertoldi, 2017). Given that cutting is scalable, kirigami principles have found applications in many engineered systems with vastly different scales, like nano/mesoscale devices (Xu et al., 2018), composite laminates (Lele et al., 2019), wearable sensors (Xu et al., 2019), and robotics (Firouzeh et al., 2020; Yang et al., 2021). In particular, kirigami skin has been combined with other soft actuators to improve the performance of crawling robots by significantly increasing the friction between the robotic body and its surrounding medium (Rafsanjani et al., 2018). Kirigami-based skin can also help precisely program the shape morphing performance of an inflatable structure (Jin et al., 2020).

In this study, the kirigami skin, wrapped outside of an inflatable, cylindrical-shaped balloon, has two groups of slit cuts with a prescribed angle between them (Figure 1B). These cuts are made on a plotter cutter or laser cutter, which is more precise, efficient, and cost-effective than other fabrication methods of soft actuators (e.g., reinforcing fiber wrapping and elastomer molding). When the internal balloon is inflated and applies pressure to the kirigami skin, the facets defined by the slit cuts start to exhibit both in-plane rotation and out-of-plane buckling deformation. As a result, the kirigami skin can transform the volumetric expansion of the internal balloon to a combined twisting and axial extension motion. More importantly, these motion characteristics can be prescribed directly by tailoring the kirigami cutting design.

Therefore, this study aims to build upon our proof-of-concept preliminary study in (Iannucci and Li, 2020) and uncover the linkage between the kirigami skin design and the corresponding actuation motion characteristics. To this end, we formulate a new kinematics model that introduces “virtual folds” to the kirigami skin at locations showing the most concentrated deformation. Assuming these folds behave like perfect hinges and the surfaces in between these folds remain flat (aka. rigid facets), one can significantly simplify the complex kirigami skin deformations into a one-degree-of freedom mechanism. Nonetheless, experimental validation shows that this kinematics model can accurately predict the actuator twisting and extension, providing a viable tool for design optimisation. In addition, we conduct additional tests to assess the pressure-stroke performance and elastoplastic properties of the kirigami-skinned actuator to formulate an operation strategy for repeatable actuation performance.

In what follows, Section 2 introduces the design and fabrication of the kirigami skinned actuator; Section 3 details the formulation and experimental validation of the new kirigami skin model; Section 4 discusses the elastoplastic properties of the kirigami actuator; and Section 5 ends this paper with a conclusion.

## 2 Design and Fabrication of the Kirigami-Skinned Actuator

The actuator’s kirigami skin has two types of uniformly distributed slits cuts, dividing the skin sheet into a tessellation of identical “unit cells” (Figure 1B). For clarity, we refer to these two types as the “horizontal cuts” and “inclined cuts” (although the inclined cuts can be orthogonal to the horizontal cuts as a special design case). It is worth highlighting that the horizontal and inclined cuts’ positions do not strictly align with the circumferential (*x*−) and axial (*y*−) axes of the cylindrical actuator. Instead, there is an offset (*d*) between adjacent cuts to facilitate the kirigami skin’s deformation under pneumatic pressure (as we detail further in the following Section 3). Denote *l*_*c*_ as the length of both horizontal and inclined cuts, *l*_*δ*_ as the “hinge” size in-between two different types of cuts, and *η* as the angle between inclined cuts and the actuator’s axial axis (Figure 1C). The un-deformed unit cell’s dimensions are

$$l_{ux}^{o}=l_{c}+2l_{\delta }\cos\eta ,$$ (1)

$$l_{uy}^{o}=2l_{\delta }+l_{c}\cos\eta .$$ (2)

The offset between adjacent cuts are *d* = 2*l*_*δ*_  sin *η*. Moreover, the number of unit cells *N*_*x*_ in the kirigami skin’s circumferential axis depends on the offset *d*, and it should roughly follow a geometric constraint:

$$N_{x}=\frac{l_{\delta }\left(1+\sin\eta \right)+l_{c}\cos\eta }{2l_{\delta }\sin\eta }.$$ (3)

In this way, as one wraps the kirigami skin around the actuator body, the slits cuts at both ends can align properly, and the corresponding undeformed actuator radius is $R^{o}=N_{x}l_{ux}^{o}/2\pi$. On the other hand, the number of unit cells in the axial direction *N*_*y*_ is a free variable, and it directly determines the pneumatic actuator’s undeformed length in that $L^{o}=N_{y}l_{uy}^{o}$. Finally, we define a non-dimensional “cut ratio” *R* as the ratio of hinge size over the slit cut length in that

$$R=\frac{l_{\delta }}{l_{c}}.$$ (4)

To fabricate the kirigami-skinned actuator, we first use an FCX4000 mechanical plotter (Graphtec, Tokyo, Japan) to slit cut a 0.5 mm thin Artus Plastic Shim Stock according to the desired kirigami design. We found that this thin Artus Plastic sheet has sufficient elastic flexibility and tear resistance to withstand the large deformation and concentrated stress induced by the out-of-plane deformation near the slit cuts. It is worth noting that we add tiny cuts at the tip of horizontal and inclined cuts to make the kirigami skin more mechanically resilient (Figure 1C). The kirigami slit cuts can act like cracks in the skin, leading to excessive stress concentration and fracture failure under pneumatic pressure, but the additional tiny cuts can significantly slow down such “crack growth,” effectively increase the kirigami skin’s toughness. Then, we connect the kirigami skin to two 3D printed end fittings via double-sided tape. To ensure reliable actuation performance, we slightly overlap the two ends of kirigami skin and bond them using double-sided tapes Figure 1B. Without such overlapping, the kirigami skin would have a discontinuous break, causing the inner balloon to bulge through under pressure. We wrap vinyl tape outside of the skin-end fitting assembly for extra support, and use 50 mm diameter balloons (Qualatex, North Wichita, KS) to supply the pneumatic pressure. Each balloon, with initial length customized according to the kirigami skin’s length, is connected to 3D-printed end cap and pre-inflated to initiate the balloon-skin contact before testing. Finally, we attach a long straight rod at the free end of this balloon (Figure 1A). This rod could move freely through the end fitting and facilitate the measurement of the actuator’s deformation under pressure.

## 3 Kinematics of the Kirigami Skin

**FIGURE 2:**
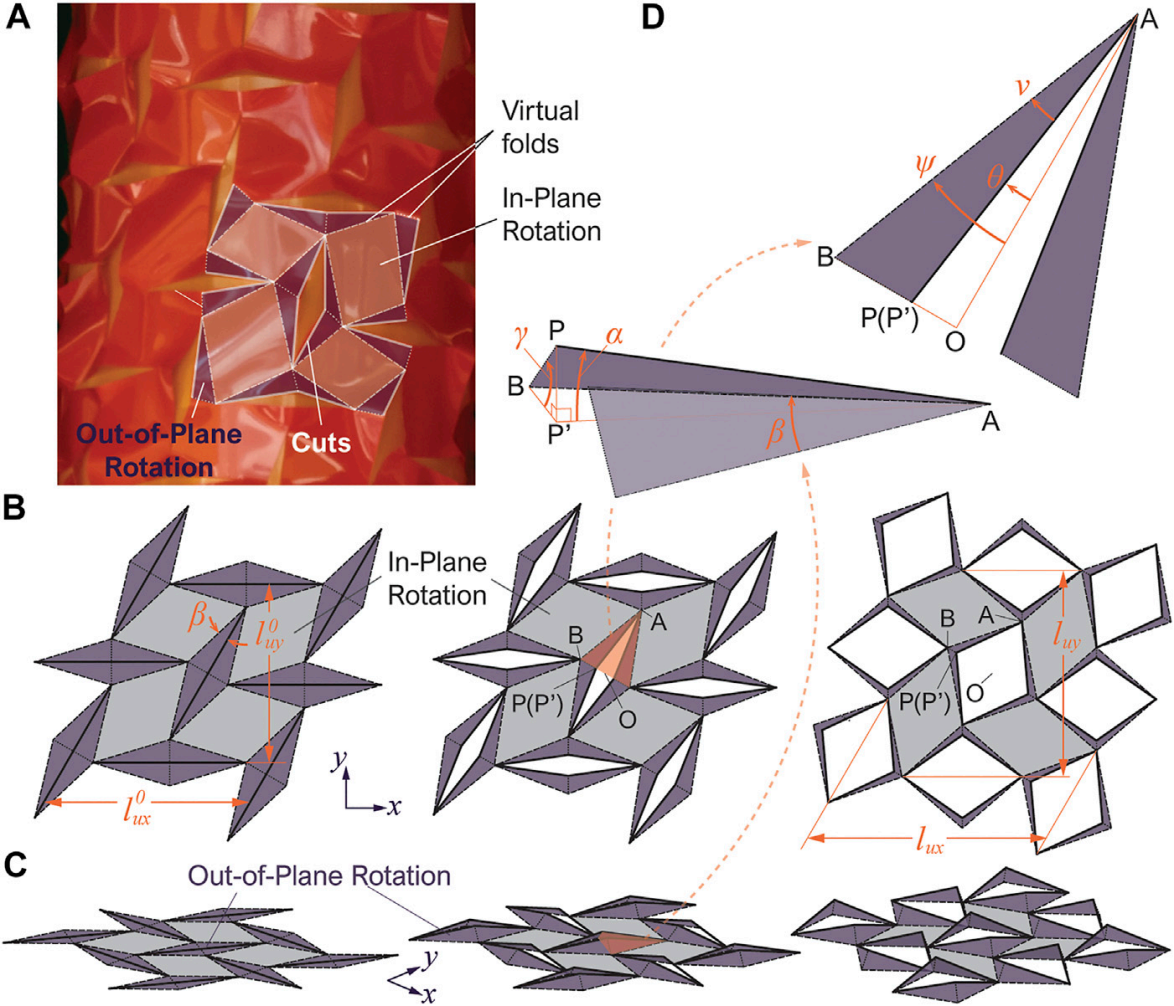
3D schematic diagrams showing the deformation of the kirigami skin based on the new kinematics model. **(A)** Another close-up view of the kirigami skin’s deformation, showing the “virtual” hinge-like folds and rigid flat facets. Here, the grey-colored parallelogram facets only rotate in-plane, while the purple-colored triangular facets rotates out-of-plane. The dashed lines are valley folds and the dotted lines are mountain folds. **(B,C)** Top view and isometric view of the unit cell’s deformation, respectively. **(D)** Close-up view of a pair of triangular facets, showing the important geometric parameters.

An important question regarding the kirigami-skinned actuator is how the cutting pattern design relates to the actuator’s overall extension and twisting motions. To this end, careful observations of the skin deformation can provide critical insights. As the actuator is pressurized, its internal balloon expands in volume and, as a result, pushes the kirigami skin from inside and also stretches it in both axial and circumferential axes. As a result, the kirigami skin is forced to deform in-plane and buckle out-of-plane. While it is well-understood that a stretched kirigami skeet can develop out-of-plane deformations (Rafsanjani and Bertoldi, 2017; Iannucci and Li, 2020), the kirigami skin’s deformation in this study is uniquely different. Due to the presence of the internal balloon, the skin can only buckle radially outwards (aka. toward only one side of the kirigami sheet), which fundamentally differs from the stand-alone kirigami sheets that buckle towards both sides. More importantly, as the out-of-plane buckling becomes significant, we observe that the kirigami sheet’s deformation starts to concentrate locally, such as in the narrow regions connecting the two ends of adjacent cuts (Figures 1E, 2A). In contrast, the surfaces between these concentrated deformation primarily exhibit rigid-body rotations without significant deformations.

Therefore, we can place *hinge-like* “virtual folds” according to the concentrated deformation region and divide the kirigami sheets into *rigid* “facets” as shown in Figure 2A. The parallelogram facets are assumed to remain in contact with the internal balloon surface and rotate in-plane only, while the triangular facets can rotate out-of-plane with respect to the hinge-like folds. These assumptions essentially simplify the complex kirigami skin deformation into a 3D linkage-like mechanism with only one degree of freedom. Therefore, we can derive closed-form solutions for the kinematics of the Kirigami skin and uncover the linkage between the cutting pattern design and the actuator’s motion characteristics. The following subsection details the mathematical formulation of a new kinematics model based on these assumptions.

### 3.1 Mathematical Modeling

To avoid unnecessary complexities, we first analyze the unit cell’s deformation and assume that this cell starts from a completely flat configuration (aka. ignoring the curvature of the cylindrical balloon surface). Figures 2B,C illustrates the unit cell’s deformation based on the virtual fold and rigid facet assumptions, and Figure 2D is a close-up view of a triangular facet that deforms out-of-plane. Denote this triangular facets as *ABP* as it is formed by two virtual folds (*AB*, *BP*) and half of a slit cut (*AP*). The length of the virtual fold *AB* is $l_{AB}={\left(l_{\delta }^{2}+l_{c}^{2}/4\right)}^{1/2}$, and the sector angle between *AB* and *AP* is *β* = tan^−1^(2*l*_*δ*_/*l*_*c*_). Now, denote *ABP*’ as the *projection* of triangular facet *ABP* onto the reference *x* − *y* place, so that *α* is the angle between *AP* and *AP*’, and *β* is the angle between *BP* and *BP*’, applying the rules of trigonometry yields

$$\frac{l_{\delta }}{l_{c}/2}=\frac{\sin\alpha }{\sin\gamma }.$$ (5)

Here, two angles can describe the rigid-body rotations of this triangular facet. They are the sector angle of the projected triangular facets *ν* = *∠P*′*AB*, and the opening angle of the kirigami slit cuts *θ* = *∠OAP*’, where *O* is the center point of the opened slit cut. Note that if the kirigami skin is un-deformed, the triangular facet *ABP* lays on the reference plane so that *ν* = *β* and *θ* = 0°. Once the kirigami skin deforms out-of-plane, one can apply the law of sines and obtain

$$\sin\nu =\frac{l_{\delta }}{l_{AB}}\cos\gamma \cos\theta .$$ (6)

Details regarding the derivation of this equation can be found in the Appendix. Once the out-of-plane rotation of the triangular facets are determined, we can use it to calculate the overall deformation of the unit cell. To this end, we observe that the orientation of slit cuts does not change during actuation: The horizontal cuts remain horizontal and the inclined cuts are still inclined at an angle *η* with respect to the actuator’s axial axis. Denote another angle *ψ* = *θ* + *ν*. Since *BO* = *BP*’ + *P*′*O*, *BO* = sin(*θ* + *ν*), *BP*’ = *l*_*δ*_  cos *γ*, and *P*′*O* = 0.5*l*_*c*_  cos *α* sin *θ*, one can obtain

$$\sin\psi =\frac{0.5l_{c}\cos\alpha \sin\theta +l_{\delta }\cos\gamma }{l_{AB}}.$$ (7)

The *deformed* unit cell’s dimension can be calculate as:

$$l_{ux}=2l_{AB}\left(\sin\psi \cos\eta +\cos\psi \right),$$ (8)

$$l_{uy}=2l_{AB}\left(\sin\psi +\cos\psi \cos\eta \right).$$ (9)

If the unit cell is un-deformed, *ψ*_min_ = *β*, and these two equations converge to Eqs 1, 2 in the previous section. Due to pneumatic pressurization, the kirigami skinned actuator will expand radially. Therefore, we can prescribe *l*_*ux*_ as the input to calculate the angles (*α*, *γ*, *θ*, *ν*) and the unit cell’s dimension *l*_*uy*_ by solving Eqs 5–9.

Once the unit cell’s deformations are known, one can use them to calculate the actuator’s axial extension and twisting. The key here is to analyze the anisotropy in the unit cell’s deformation in the circumferential (*x*−) and axial (*y*−) axes. To this end, we define a reference angle *ϕ* to describe the aspect ratio of the *un-deformed* unit cell in that

$$\phi =\tan^{-1}\left(\frac{l_{ux}^{0}}{l_{uy}^{0}}\right).$$ (10)

If the inclined cuts are perpendicular to the horizontal cuts (aka. *η* = 0°), the unit cell expands in *x* and *y*−axes without changing its aspect ratio so that *l*_*ux*_ = tan *ϕl*_*uy*_. As a result, the overall kirigami skin exhibits bi-directional in-plane expansion without any shearing, creating an axial extension in the actuator without twisting.

However, when the incline angle *η* is not zero, two changes occur. First, the unit cell’s expansion becomes anisotropic, and in particular, the cell deforms more in the circumferential *x*−axis than in the axial y-direction so that its aspect ratio changes. We denote such “excessive” deformation in the *x*−axis as

$$\Delta l_{ux}^{e}=l_{ux}-l_{uy}\tan\phi .$$ (11)

The second change from a non-zero incline angle *η* is that the positions of adjacent unit cells will have an offset, which is the same as the slit cuts’ position offset *d* discussed in the previous section. As a result, the kirigami skin exhibits shearing deformation and creates a twist in the actuator. The twisting angle of the free end of this actuator is

$$\Omega =\frac{N_{y}\Delta l_{ux}^{e}}{R},$$ (12)

where *R* is the pressurized actuator’s radius

$$R=\frac{N_{x}l_{uy}\tan\phi }{2\pi }.$$ (13)

Finally, the kirigami skinned actuator’s axial extension, or extended length, is simply

$$E=N_{y}l_{uy}.$$ (14)

It’s worth noting that our kirigami kinematics model also predicts the maximum admissible expansion in *l*_*ux*_. By solving *∂l*_*ux*_/*∂ψ* = 0, we find that the maximum deformation occurs when

$$\psi =\psi_{\text{max}}=\tan^{-1}\left(\cos\eta \right).$$ (15)

### 3.2 Experiment Validation

**FIGURE 3:**
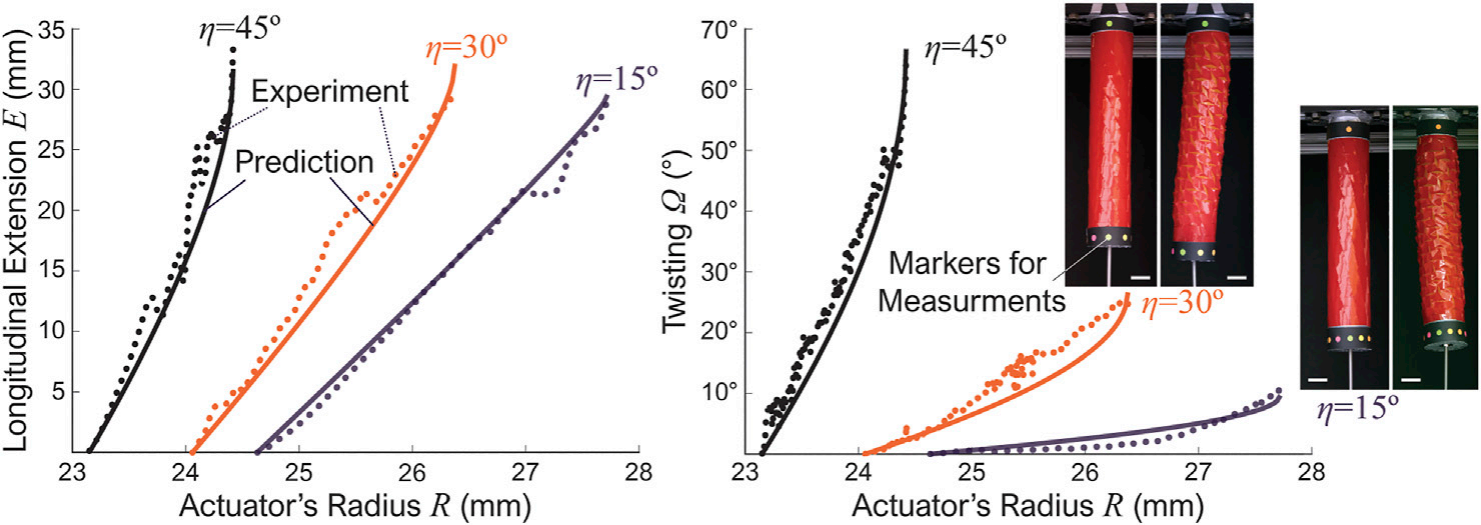
Experiment results from three kirigami-skinned actuator prototypes with different incline angles *η*. The dotted lines are readings from the experiment and solid lines are the kinematics model predictions. The scale bars in the pictures are approximately 2 cm.

To experimentally validate the kirigami kinematics model, we fabricate and test three actuator samples with the same cut and hinge length (*l*_*c*_ = 20 mm and *l*_*δ*_ = 3 mm) but different incline angles: *η* = 15°, *η* = 30°, and *η* = 45°. To measure the actuator’s deformation, we hang them vertically on a custom-built test frame and attach color markers at their free ends (Figure 3). As we gradually increase the internal pressure, high-resolution videos were captured to record the actuators’ deformation. We then use MATLAB’s image processing programs to measure the actuators’ diameter and the end fittings’ axial/rotational displacements.

Figure 3 summarizes the experiment results and the corresponding kinematics model predictions. Notice that, although the Kirigami skins of three actuator samples have the same number of unit cells (*N*_*x*_ = 6, *N*_*y*_ = 8), their initial radius are different due to different inclined slit cut angles. It is also worth noting that the design of three prototypes only approximately follow Eq. 3, but the kirigami skin still overlap well during fabrication. Overall, there are minor discrepancies between the experiment and theoretical results, probably due to the fabrication errors and imperfect contact between the balloon and kirigami skin. However, the general agreement is quite well, so these test results validate our assumptions underpinning the kirigami skin’s kinematics model.

In addition, there are two interesting observations from these results. First, the actuator’s axial extension and twisting are almost linearly related to the radial expansion initially. However, these correlations become significantly nonlinear as the parallelogram facets’ in-plane rotation and triangular facets’ out-of-plane rotation increase. Such geometric non-linearity will be a critical factor for future robotic manipulation control. Secondly, the incline cut’s angle *η* has a strong influence on the actuator’s performance. That is, as the inline angle *η* increases, the twisting also increases significantly. This observation indicates that we have considerable freedom to prescribe or “pre-program” the actuator’s overall performance by simply tailoring to the kirigami cut designs, as we detail further in the following subsection.

### 3.3 Kirigami Design Parametric Study

**FIGURE 4:**
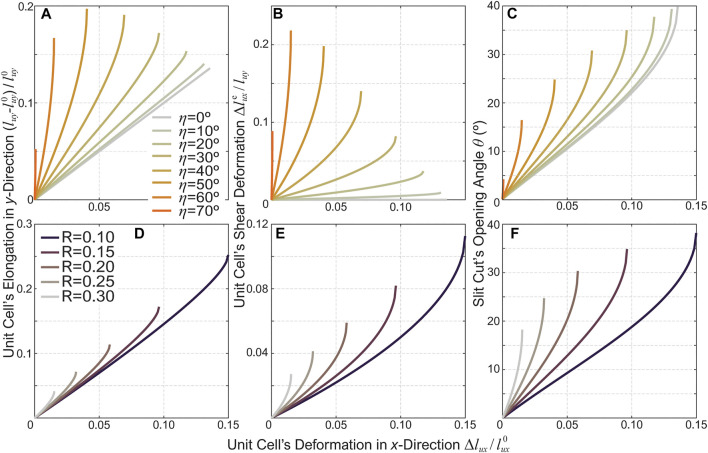
Parametric analysis results showing the correlation between the kirigami cutting pattern design and the actuator’s motion characteristics. The first row **(A-C)** shows the results from kirigami skins with the same cut ratio (*R* = 0.15) but different incline angles, while the second row **(D-F)** shows the results based on the same incline angle (*η* = 30°) but different cut ratios. The left column **(A,D)** shows the normalized unit cells’ axial expansion, the middle column **(B,E)** shows the unit cells’ shearing, and the right column **(C,F)** show the opening angle *θ* of the slit cuts.

To further reveal the correlations between the Kirigami cutting pattern design and the actuator’s deformation. We conduct further parametric analyses based on the validated kinematics model. Figures 4A-C show the *normalized* unit cell axial elongation, shearing, and slit cut’s opening angle with the same cut length *l*_*c*_, hinge size *l*_*δ*_, but different incline cut angles *η*. Here, the unit cell’s axial elongation is directly related to the actuator’s extension according to Eq. 14, while the unit cell’s shearing dictates the overall twisting based on Eq. 12. The end of each curve in these figures corresponds to the maximum radial expansion *ψ*_*max*_ (Eq. 15). Generally speaking, a higher *η* angle gives a more significant twisting. On the other hand, there exists an optimal angle to obtain the maximum extension. We find this angle is *η*_max*E*_ = cos^−1^(tan *β*).

Moreover, Figures 4D-F shows the actuators’ motion characteristics based on the same incline angle *η* = 30° but different cut ratios *R* according to Eq. 4. The cut ratio also has substantial influences on the actuator’s motion. A kirigami skin with a smaller cut ratio can achieve more considerable deformation (extension and twisting), because the hinge size *l*_*δ*_ is relatively smaller than the cut length *l*_*c*_. However, one must also consider the elastoplastic behaviors of the kirigami actuator when selecting the cut ratio, as we discuss briefly in the following section.

It is worth noting that, compared to other soft pneumatic actuators, especially those based on pneumatic chambers without any reinforcements, the kirigami-skinned actuator might have a smaller deformation. So there is likely a trade-off between maximum stroke performance and fabrication simplicity. Fortunately, one has the freedom to fine-tune the cut ratio and increase the stroke according to potential application requirements.

## 4 Pressure-stroke Relationship of Kirigami-Skinned Actuators

After investigating the kinematics of the kirigami skinned actuator, we turn our focus to the mechanics in this section. In particular, we examine the free stroke performance and elastoplastic behaviors (e.g., hysteresis and performance repeatability under cyclic loading), which are crucial for practical applications of the pneumatic actuators in different applications. To this end, we test the pressure-stroke relationship of the pneumatic actuators with different kirigami skin designs and under different loading sequences. It is worth emphasizing that, to avoid unnecessary complexities, we use skins exhibiting axial extension only (aka. *η* = 0°). However, the results and insights from the following mechanics analysis certainly apply to twisting kirigami skins with non-zero incline angles. It is also worth noting that only the pressurization (or extension) part of the loading cycles are reported in this section.

**FIGURE 5:**
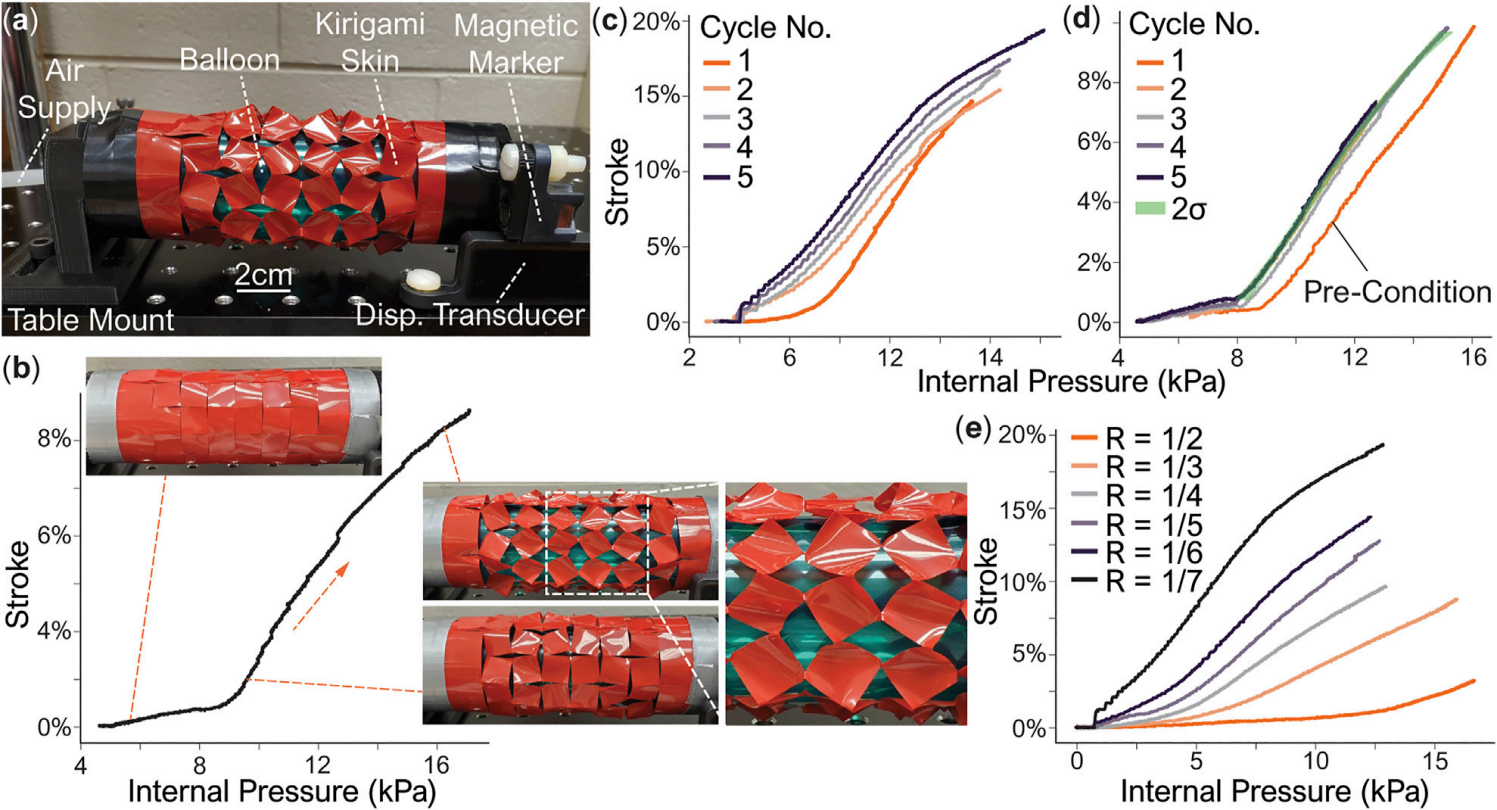
Free stroke testing results from the kirigami-skinned actuators. **(A)** The test setup. **(B)** A pressure-stroke curve of a prototype with *R* = 1/6 and the corresponding skin deformation pattern. The two deformation phases are evident. **(C)** Cyclic loading responses from this prototype. Each loading cycle achieves a higher stroke than the previous one. **(D)** Another cyclic loading result. In this case, the prototype is pressurized to 10% stroke in the first pre-condition cycle, and then used with a smaller stroke in the subsequent cycles. The standard deviations of Cycles 2-5 are highlighted, showing a repeatable performance. **(E)** The measured performance of actuator prototypes with different cut ratios. Experiment images adapted from Ianucci and Li, 2020 with permission.

Figure 5A shows the test setup. To measure the free stroke of these actuators, we mount one of the actuator’s end fittings to a non-ferrous bed while leaving the other end fitting free. A non-contact electromagnetic linear displacement transducer (SPS-L075-HALS, Honeywell, Charlotte, NC) was mounted to the bed, with the magnetic positional marker secured to the free-moving end fitting. Pneumatic pressure is supplied using a solenoid valve (ControlAir 900X, Amherst, NH) and measured by a gauge pressure sensor (SSCDANN005PGAA5, Honeywell, Charlotte, NC). Once the actuator reaches full extension, we release the pressure by a manual relief push-button valve, which returns the actuator to its initial length. The pressure sensor and displacement transducer readings are correlated using LabView via an analog data acquisition instrument (National Instruments, Austin, TX).

Figure 5B displays the pressure-free stroke curve of an actuator during its pressurization (or extension), which shows two distinct phases. Here, the free stroke is calculated as

$$\text{Free Stroke}=\frac{E-L^{o}}{L^{o}}=\frac{N_{y}l_{uy}-N_{y}l_{uy}^{o}}{N_{y}l_{uy}^{o}}.$$ (16)

In the first phase, at low pressure, the kirigami skin shows minimal stroke. In this phase, the kirigami skin deforms primarily in-plane. However, once the pressure reaches a critical value, the kirigami skin enters the second phase and begins to deform both in-plane and out-of-plane significantly (according to the kinematics model discussed in the previous section (see the insert, close-up view in Figure 5B). As the pressure continues to increase, the parallelogram and triangular facets in the kirigami skin continue to rotate until they reach the maximum kinematic deformation defined in Eq. 15.

Plastic deformation plays a important role in the nonlinear response of kirigami skin. Figures 5C,D shows the pressure-stroke relationships of two actuator samples with the same cutting pattern design but different cyclic loading sequences. On the first actuator sample, we apply five cyclic loading cycles. In each cycle, we increase the pressure until this actuator reaches a new maximum stroke that has not been achieved before. Due to the progressively increasing plastic deformations, the kirigami skin shows less resistance against pressure over the loading cycles, thus providing better performance in terms of the free stroke at a given pressure. Therefore, to create an actuator with consistent and repeatable performance over many pressurization cycles, the kirigami actuator must be pressurized to its maximum strain without failure as a “pre-conditioning” procedure before practical implementation. For example, for the second actuator sample, we first pressurize it until it reaches a 10% free stroke, and then restrict the operating stroke to be less than 10% in the following loading cycles (Figure 5D). As a result, the actuator performance becomes reliably repeatable. Such repeatability is due to the fact that the kirigami sheet has been plastically deformed as much as possible without failure so that no new plasticity can occur in the following loading cycles.

Similar to the kinematics analysis, the pressure-stroke relationship of the kirigami actuator is closely related to the underlying cutting pattern design. For example, a smaller cut ratio *R* in the kirigami skin gives a lager free stroke at the same pressure level (Figure 5E). This is because the smaller hinges corresponding to the smaller cut ratio can accommodate more significant facet rotations. In our test, we achieved close to 20% of free stroke with *R* = 1/7. On the other hand, kirigami skins with a larger cut ratio (or large hinge size) can withstand higher internal pressure.

## 5 Summary and Conclusion

This study establishes a design and analysis framework for constructing kirigami-skinned pneumatic actuators with pre-programmable actuation capability. In particular, we examine the nonlinear kinematics and mechanics characteristics of kirigami skins consisting of two groups of slit cuts in different orientations. When the actuators is pressurized internally, its kirigami skin deform anisotropically due to the slit cuts, converting the volumetric expansion to a combination of axial extension and twisting motions. Such extension and twisting can be prescribed by simply tailoring the angle and length of these slit cuts. To this end, we formulate and experimentally validated a new kinematics model to accurately describe the correlations between cutting pattern design and overall actuators’ motion. This model uses a virtual crease and rigid-facet assumption to simplify the complex kirigami skin deformation into an equivalent 3D linkage mechanism without significantly sacrificing accuracy. Therefore, it can identify the optimal kirigami cutting pattern design according to the actuation performance requirements.

Besides kinematics analysis, we also tested the pressure-free stroke performance of actuators with orthogonal cuts with different cut ratios. We found that the pressurized actuators undergo two phases of deformation. The kirigami skins exhibit small deformations in-plane at the low-pressure phase, so the actuator’s stroke is minor. However, once the pressure reaches a threshold, the kirigami skin enters the second phase and starts to deform significantly out-of-plane according to the kinematics model predictions. The kirigami skinned actuator also exhibits elastoplastic behaviors. We found that, for reliable and repeatable actuation performance, one should pre-condition the kirigami skin by stretching it to the maximum deformation without failure before practical applications.

This paper aims to presents the concept of kirigami-skinned actuator and elucidate the underlying principles between cutting patterns and actuator deformation. Certainly further studies are necessary, such as an in-depth analysis of the load capacity (aka. block force), hysteresis, and further refinement of fabrication process to ensure the actuators’ durability. These will be vital topics for follow-up studies. Regardless, the results of this study overall can open up a new approach to construct versatile and simple-to-fabricate pneumatic actuators—with sophisticated motion capabilities—for soft robotic applications.

## Data Availability

The original contributions presented in the study are included in the article/Supplementary Material, further inquiries can be directed to the corresponding author.

## Appendix

In the right triangle Δ*AOB*, the angle *∠ABO* is *π*/2 − (*θ* + *ν*) and *∠AP*′*O* in the triangle Δ*AOP*′ is *π*/2 −*θ*; therefore, *∠AP*′*B* is *π*/2 + *θ*. Thus, by applying the sine law to triangle Δ*AP*′*B*, Eq. 6 can be obtained. Additionally, by substituting *BO* = sin(*θ* + *ν*), *BP*’ = *l*_*δ*_  cos *γ*, and *P*′*O* = 0.5*l*_*c*_  cos *α* sin *θ* into *BO* = *BP*’ + *P*′*O*, Eq. 7 holds true.
